# An effectively protective VLP vaccine candidate for both genotypes of feline calicivirus

**DOI:** 10.3389/fimmu.2024.1515342

**Published:** 2024-12-20

**Authors:** Yupeng Yang, Zhe Liu, Mengru Chen, Kexin Feng, Ruibin Qi, Hongtao Kang, Qian Jiang, Liandong Qu, Jiasen Liu

**Affiliations:** ^1^ State Key Laboratory for Animal Disease Control and Prevention, Harbin Veterinary Research Institute, Chinese Academy of Agricultural Sciences, Harbin, China; ^2^ College of Veterinary Medicine, Northeast Agricultural University, Harbin, China

**Keywords:** feline calicivirus, genotypes, virus-like particles (VLPs), neutralization titers, challenge trials

## Abstract

Feline calicivirus (FCV) is one of the most widespread pathogens affecting feline animals. Currently, FCV is believed to be divisible into two genotypes, with prevalent strains encompassing both GI and GII. Vaccination is the primary means of preventing FCV infection, yet traditional inactivated or attenuated vaccines theoretically pose potential safety concerns. In this study, a virus-like particles (VLPs), named DL39-VLPs, was constructed, using the *VP1* gene of FCV DL39 strain with broad neutralizing and protective properties as a template through insect cell expression system. Cats were immunized with a 50 μg dose of DL39-VLPs mixed with an oily adjuvant via subcutaneous injection in the neck. For feline antisera positive for DL39-VLPs, the titer range in neutralization tests against prevalent GI strains ranged from 1:151 to 1:538, whereas the titer range in neutralization tests against prevalent GII strains was between 1:65 and 1:113. Challenge trials demonstrated that cats immunized with DL39-VLPs exhibited no significant clinical symptoms and had significantly reduced viral shedding and viremia compared to the challenged control group. Ultimately, a safe and highly protective VLP vaccine candidate, DL39-VLPs, was developed, which provides an important tool for the prevention and control of FCV infection.

## 1 Introduction

Cats is one of the closest companion animals to humans, whose health has garnered increasing public attention. They remain susceptible to viral infections, with feline calicivirus (FCV) standing out as one of the most pervasive pathogens posing a considerable threat to feline animals. FCV, a member of the Vesivirus genus in the Caliciviridae family, possesses a positive-sense, single-stranded RNA genome, roughly 7700 nucleotides long (1). The genome comprises three open reading frames (ORFs), among which the *ORF2* gene is responsible for encoding the major capsid protein VP1 and is further subdivided into six regions, labeled A to F, and serves as the primary antigenic region of FCV (2). Up to this point, only a singular serotype of FCV exists, yet multiple studies indicate the existence of two primary genotypes (3, 4). Typically, FCV leads to upper respiratory illnesses in felines, predominantly manifesting as oral ulcers, with certain cats experiencing lameness, abortion, skin ulcers, and potentially systemic conditions resulting in fatality (5–8). In recent years, the prevalence of FCV in China has gradually increased, with outbreaks reported in multiple regions (9, 10, 11).

Presently, the primary immunizations against FCV consist of vaccines with attenuated live and those that are inactivated. In European countries, the attenuated live vaccine of F9 and other vaccine is employed for FCV prevention, and recent epidemiological studies have shown that the F9 strain can neutralize the majority of FCV strains (12, 13). In China, both domestically developed as well as imported, the vaccines against FCV belong to the category of inactivated vaccines. However, inactivated vaccines exhibit certain drawbacks, including the possibility of incomplete viral inactivation or the unintended alteration of crucial antigen structures during the inactivation procedure. Consequently, the creation of safer vaccines is of paramount importance for mitigating the infection risks associated with the employment of inactivated vaccines in manufacturing or clinical immunization programs, while also augmenting vaccine immunogenicity. Virus-like particle vaccines (VLPs), given their structural resemblance to viruses, are deemed a highly promising class of protective vaccines.

Crafting VLPs through the *Spodoptera Frugiperda* (*Sf9*) cells expression system is a recognized method, yet literature on the effective creation of VLPs for FCV is scarce. Certain researchers have employed rabbits for creating immune sera targeting FCV-VLPs, discovering their capacity to produce potent neutralizing antibodies against FCV in rabbits (14). However, a comprehensive evaluation of FCV VLPs in the natural host of cat is still not documented. This study utilized the *VP1* gene from the earlier examined DL39 strain, which had verified as its extensive protective properties in various biological and animal experiments, to create VLPs, termed DL39-VLPs. The immunization of the host animals further confirmed the vaccine’s superior cat protection and its capability to simultaneously shield against two distinct genotypic strains. To sum up, this work is of great important in identifying more secure vaccine variants to prevent and control FCV prevalence in China.

## 2 Materials and methods

### 2.1 Cells, plasmid and viruses

*Sf9* cells and Crandell’s Cat Cell (CRFK) are stored in our laboratory; DL31, DL38, DL39, HRB48, TIG-1 and FB-NJ-13 strains are stored in our laboratory, the specific information of them is presented in **Table 1** (4); pFastBac™ HT plasmid is preserved by the laboratory.

**Table 1:** Profiles of some strains used in this study.

FCV Strains	GenBank ID	Acquisition Time	Genotype	Host	Animal Regression Test
DL31	MW804427	2020(CHN)	GI	Cat	The body temperature of the test cat increased
DL38	MW804429	2020(CHN)	GII	Cat	Isolated from healthy cats and no regression test was performed
DL39	MW804430	2020(CHN)	GI	Cat	The cats had high body temperatures and were depressed
HRB48	MW804434	2020(CHN)	GI	Cat	All the cats showed symptoms such as sneezing, elevated body temperature, cough, mouth ulcer and plantar cracking
FB-NJ-13	KM111557	2013(CHN)	GII	Cat	All the cats showed symptoms of sneezing, elevated body temperature, cough, increased secretion of eye and nose, anorexia, tremor of limbs, severe oral ulceration and plantar dehysis
TIG-1	KU373057	2014(CHN)	GI	Tiger	The VSD Strain(Causing the death of the experimental cat)

### 2.2 Construction of DL39-VLPs recombinant shuttle plasmid

Amplification of the *VP1* gene (B-F region) was achieved with the DL39 strain’s cDNA stored in the lab, and the pFastBac™ HT plasmid underwent simultaneous digestion with *Eco*R I and *Hin*d III enzymes, followed by gel electrophoresis. After retrieving the target bands, the B-F fragment was reattached to the plasmid via homologous recombinase, then transferred into DH5α competent cell, post-selection and cultivation of the colony for growth, the plasmid was extracted. The plasmids that were created with success were designated as pDL39-VLPs. Subsequently, the pDL39-VLPs underwent transformation into DH10Bac competent cell to screen for blue-white spots, selecting one white-spotted colony for growth, and conducting three successive streak cultures, which were then identified using M13F/R. Only a single band was the desired bacterial fluid and the rod was extracted.

### 2.3 Generation of DL39-VLPs recombinant baculovirus

Initially, the shuttle plasmid (rod) concentration was measured, followed by the distribution of *Sf9* cells into a 6-well cell culture plate and their transfection upon reaching 70% growth, adhering to the guidelines of the Cellfectin II transfection agent. Following a 4-hour transfection, the medium was switched to a complete one, and the cell condition was monitored every 12 h. Observing clear cell lesions, the cell cultures were gathered, the supernatants centrifuged, and the new cells were inoculated with the viral solution at a ratio of 1:100. After three consecutive infections, the cell supernatant was collected and the viral titer was determined, and stored at 4°C.

### 2.4 Biological characterisation of DL39-VLPs

The specific expression of DL39-VLPs was detected using Western blot (Wb) and indirect immunofluorescence assay (IFA). Suspended *Sf9* cells were taken and infected with recombinant baculovirus with MOI = 1. Supernatants from infected cells were gathered at intervals of 24, 48, 72, 96 and 120 h. Concurrently, the TCID_50_ value of the recombinant baculovirus was determined using the end-point dilution method, represented as a growth kinetic curves, and the VP1 protein expression was concurrently measured. Ultimately, DL39-VLPs were abundantly produced in ideal conditions, and VLPs underwent purification through sucrose concentration gradient centrifugation, followed by electron microscopic observation.

### 2.5 Determination of feline anti-DL39 VLPs sera neutralization titers

The protein concentration of purified DL39-VLPs was determined, 15 Chinese field cats, each 2 months old and negative for FCV antibodies, were bred and segmented into five groups. Initially, two groups were chosen for vaccination through subcutaneous inoculation at a 50 μg dose per, combined equally with Montanide™ GEL adjuvant, while three control groups cats inoculated an identical adjuvant dose and DMEM. After immunization, the neutralization titers of the antisera from the immunized group against prevalent strains of genotype GI (DL31, DL39, HRB48 and TIG-1) and genotype GII (DL38 and FB-NJ-13) were determined.

### 2.6 Evaluation of the immunological efficacy of DL39-VLPs

Continuing from the aforementioned test, the immunization challenge trials were subsequently conducted. Both cat groups vaccinated with DL39-VLPs received HRB48 (GI) and FB-NJ-13 (GII) injections at a rate of 1 × 10^8^ TCID_50_ per cat. Concurrently, a positive control group was established for the same dosage of HRB48 and FB-NJ-13 tapping, while the other group was used as a negative control. In the following fortnight, the clinical performance of the cats in each group was carefully observed daily and scored according to a uniform scoring criterion, and their body temperature, body weight, detoxification status, viraemia and histopathological changes were also continuously monitored. Throughout the immunization challenge trials, euthanasia was executed as a compassionate measure to terminate the experiment promptly, thereby mitigating the duration of animal suffering. This decision was taken when the animal’s body weight declined by more than 25% from its initial weight or irreversible, severe symptoms manifested. Upon the conclusion of the experimental procedures, euthanasia was administered to the experimental cats. An intramuscular injection of Zoletil 50 at a dosage of 5 mg/kg was administered to the cats, which, according to the aforementioned dosage, could effectively induce anesthesia lasting approximately 20 minutes. Subsequent to verifying the animals’ complete anesthesia, a dose of 2 mg/kg KCl solution was administered intravenously. The experimental cats were confirmed deceased upon the observation of pupil dilation, indicating the successful completion of the euthanasia process. The experiments were performed using protocols that were approved by the Harbin Veterinary Research Institutional Animal Care Committee and our experimental operations were conducted in accordance with the ARRIVE Essential 10 guidelines (15).

### 2.7 Histopathology examination

Lung tissues were excised from cats belonging to different groups for the purpose of observing pathological changes. Subsequently, tissue samples of 1 cm³ in volume were collected and subjected to overnight fixation using formalin. Following fixation, the tissues underwent slicing, paraffin embedding, and staining with the hematoxylin and eosin (H&E) method. Ultimately, the stained sections were meticulously observed and analyzed using an optical microscope.

### 2.8 Statistical analysis

All data were replicated at least three times, and statistical analysis was performed using GraphPad Prism 8.0 software. Comparisons were made using either *t*-test or two-way ANOVA. Specifically, ‘ns’ denotes no significant difference, ‘*’ indicates a significant difference with p<0.05, ‘**’ represents a highly significant difference with p<0.01, and ‘***’ signifies an extremely significant difference with p<0.001.

## 3 Results

### 3.1 Construction and Identification of DL39-VLPs

**Figure 1:**
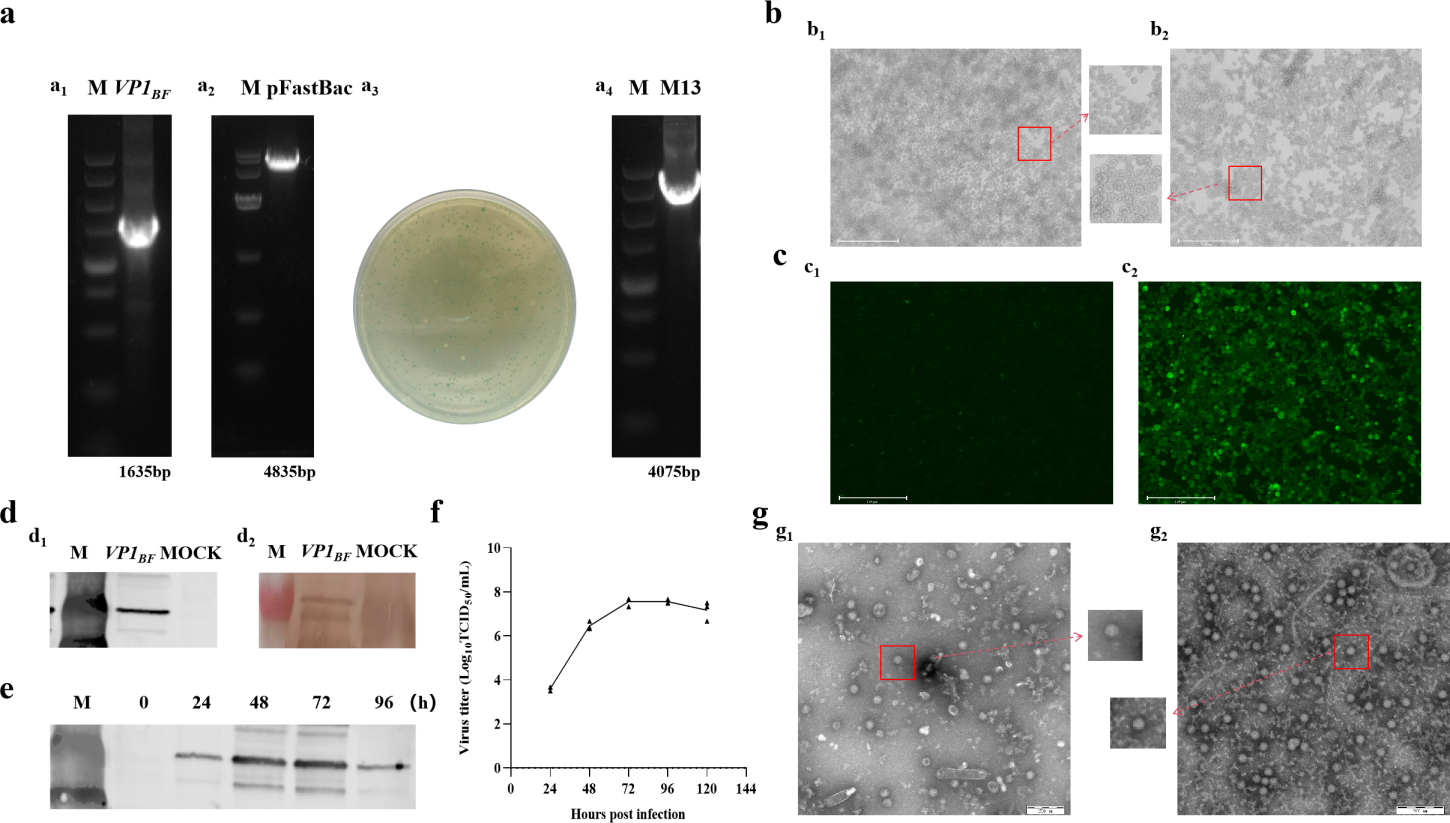
The preparation and validation of DL39-VLPs. **(A)** Preparation of recombinant plasmids for DL39-VLPs (a_1_: Amplification of *VP1_BF_*; a_2_: Plasmid digestion; a_3_: Blue-white colony screening; a_4_: Plasmid identification). **(B)** Cultivation of DL39-VLPs in *Sf9* cells (b1: Normal cells; b2: Pathological cells). **(C)** IFA Identification of DL39-VLPs (c_1_: Control group; c_2_: Experimental group). **(D)** Validation of Wb after DL39-VLPs expression (d_1_: Identification using His antibody; d_2_: Identification using feline anti-DL39 sera). **(E)** Determination of optimal sample collection time. **(F)** Determination of virus titer in different time. **(G)** Electron microscope observation of DL39-VLPs (g_1_: The morphology of DL39-VLPs; g_2_: The morphology of normal DL39 virions).

This research involved expressing FCV virus-like particles, DL39-VLPs, *in vitro* through *Sf9* insect cells, followed by an initial assessment of their immunological effectiveness. Initially, the B-F region of DL39 *VP1* gene was enlarged and effectively integrated into the pFastBac HT vector, following the screening and validation of the blue and white spots, the acquisition of the intended rod was achieved (**Figure 1A**). Subsequently, the rod was effectively transferred into *Sf9* cells, where cells infected with recombinant baculovirus exhibited a notable CPE, characterized by enlarged cell and nucleus diameters (**Figure 1B**). The IFA results, employing anti-DL39 cat serum as the primary antibody, unequivocally demonstrated the presence of pronounced green fluorescence within cells infected with the baculovirus (**Figure 1C**). Wb assay outcomes, employing both His antibody and anti-DL39 cat serum as primary antibodies, demonstrated the effective expression of *VP1* (**Figure 1D**). The assessment of viral titre in supernatants gathered at various intervals showed the peak viral titre, TCID_50_/mL=3.16 × 10^8^, 72 h post recombinant baculovirus infection, succeeded by a steady stabilization of the viral titre (**Figure 1F**). Additionally, the Wb result indicated that *VP1* expression also peaked at 72 h post infection (**Figure 1E**). Subsequent to being purified using sucrose gradient centrifugation and observation electron microscopy, the virus-like particles were packaged into nearly spherical ortho icosahedra inside the infected *Sf9* cells. The edges of these viral particles were unevenly formed and consistently sized, with diameters varying around 40 nm, closely mirroring the dimensions of actual viral particles. Even though two distinct particle types primarily formed, they lacked nucleic acid and manifested as vacant shells in contrast to actual virus particles (**Figure 1G**).

### 3.2 Results of DL39-VLPs immunization and challenge test

**Figure 2:**
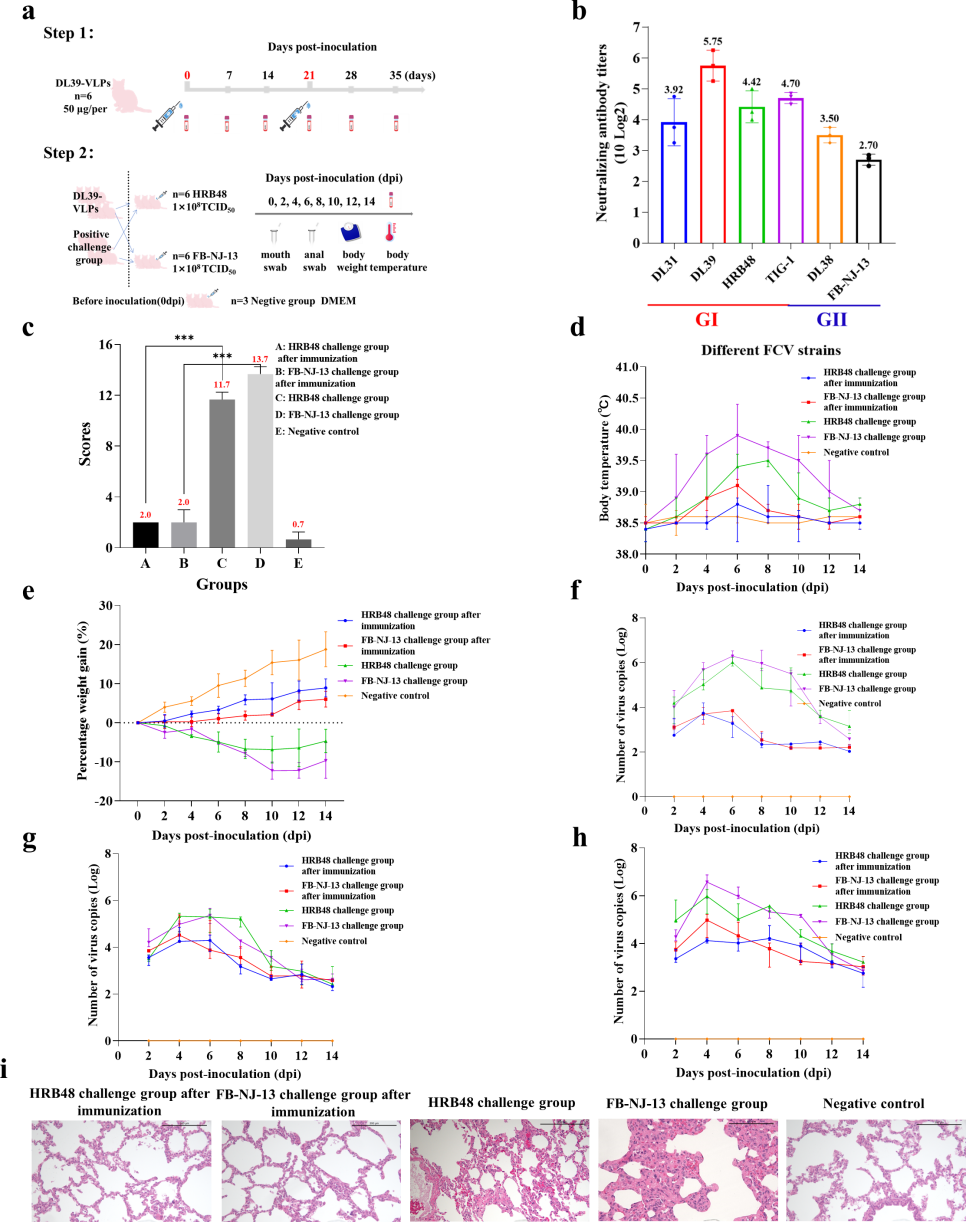
Immunization and Challenge Test of DL39-VLPs. **(A)** Schematic representation of the immunization and challenge experimental protocol. **(B)** The neutralization titers of feline anti-DL39-VLPs positive sera against prevalent strains of different genotypes. **(C)** Scoring of clinical symptoms. **(D)** Statistical analysis of body temperature. **(E)** Statistical analysis of body weight. **(F–H)** Detection of viral loads in nasopharyngeal swabs, anal swabs, and blood samples. **(I)** Histopathological analysis of lung tissues.

DL39-VLPs were prepared as immunogens and used to immunize Chinese domestic cats before conducting challenge experiments with different genotypes of FCV (**Figure 2A**). Feline anti-DL39-VLPs positive sera exhibited neutralization titers ranging from 1:151- to 1:538 against the prevalent strains of genotype GI and neutralization titers ranging from 1:65- to 1:113 against the prevalent strains of genotype GII (**Figure 2B**). The challenge results with HRB48 (GI) showed that the clinical scores of the immunized cats were significantly lower than those of the challenged group (p<0.001), but did not differ significantly from the negative control group. The challenged cats reached a body temperature of 39.5°C at 8 dpi, with weight loss, extremely high viral shedding, and severe viremia. In contrast, the immunized cats exhibited no obvious clinical symptoms and had significantly reduced viral shedding and viremia (**Figures 2C–H**). The challenge results with FB-NJ-13 (GII) showed that the clinical scores of the immunized cats were significantly lower than those of the challenged group (p<0.001), but did not differ significantly from the negative control group. The challenged cats reached a body temperature of 40°C at 6 dpi, with a significant decrease in body weight over time, extremely high viral shedding, and severe viremia. Conversely, the immunized cats exhibited no obvious clinical symptoms and had significantly reduced viral shedding and viremia (**Figures 2C–H**). Histopathological analysis further demonstrated that the lungs of the immunized cats and the negative control group remained unchanged, while the HRB48-challenged cats exhibited mild thickening of the alveolar walls, and the FB-NJ-13-challenged cats displayed mild to moderate thickening of the alveolar walls (**Figure 2I**).

## 4 Discussion

The use of inactivated vaccines is acknowledged as a crucial tactic in managing various viral infectious diseases, owing to their comparative safety and simplicity in preparation. Nonetheless, the limitations of inactivated vaccines are also evident, including the possibility of incomplete inactivation or decreased immunogenicity, which may undermine vaccine effectiveness. When administering live attenuated vaccines, it is imperative to maintain vigilance not only against potential virulence reversions and incomplete detoxification, but also against contamination by other pathogenic microorganisms. In the prevention and control of FCV, the main reliance is still on attenuated and inactivated vaccines, which could potentially lead to the aforementioned issues, thus resulting in avoidable biosafety incidents, underscoring the necessity to seek alternative vaccines to conventional ones. Relatively speaking, certain innovative vaccines have the potential to eliminate the biosafety hazards, and VLPs vaccine is an excellent alternative.

As a novel vaccine variant, VLPs lack viral genetic material, rendering them a reliable and efficient choice for vaccines against viral infectious diseases (16). Currently, more than 100 VLPs originated from human, animal, plant and bacteriophage viruses have been constructed, classified into 35 families, and are widely used in the control of viruses (16). In particular, numerous vaccines targeting VLPs against Norovirus have been effectively created, offering valuable reference for the containment of other calicivirus outbreaks (17, 18). This research employed the *VP1* gene from the DL39 strain, known for its broad protective effect against endemic FCV strains, and effectively engineered DL39-VLPs virus-like particles through the insect cell expression system, capable of producing high viral concentrations and apt for mass production. Given the FCV as a single-stranded positive-stranded RNA virus with a high degree of genetic variability, genetic evolutionary studies have concluded that there are two dominant genotypes of the prevalent FCV: GI and GII (3, 4). Consequently, in the creation of an FCV vaccine, it’s crucial to ensure its efficacy in resisting FCV infection across both genetic types. DL39-VLPs demonstrate remarkable immune response, and cats immunized with DL39-VLPs can generate substantial amounts of serum-based antigen-specific neutralizing antibodies, proving effective against both GI and GII virus genotypes. Research on animals additionally verified the efficacy of cats vaccinated with DL39-VLPs in protecting against FCV strains HRB48 (GI) and FB-NJ-13 (GII).

Despite our successful demonstration that the virus-like particles (VLPs) developed can effectively preclude infection by feline calicivirus (FCV), unfortunately, our results align with those of current inactivated and attenuated vaccines in their inability to inhibit host virus excretion. This poses a formidable challenge and underscores the need for further research to develop a newer generation of safer vaccines. Additionally, it highlights that controlling the dissemination and infection of FCV may necessitate not merely vaccination but also the augmentation of environmental hygiene control measures and the exploration of potential antiviral therapies. Moreover, VLPs have great potential for application in a variety of fields such as drug delivery, medical imaging, biocatalysis, etc. due to their numerous advantages, such as self-assembled nanoscale structures, repetitive surface epitopes, ease of genetic and chemical modification, multifunctionality as an antigen-presenting platform, and intrinsic immunogenicity (16). The application of DL39-VLPs goes beyond the development of a safer vaccine, as it provides a key way to combat other viral diseases in felines.

In summary, we developed and initially assessed DL39-VLPs, a virus-like particle vaccine derived from DL39’s *VP1* gene, designed to protects cats from various FCV genotypes. DL39-VLPs not only significantly reduces biosafety risks to the environment, but also possesses a high degree of practical value. Furthermore, it establishes the groundwork for creating additional products derived from VLPs, thereby offering technical assistance to enhance the management of cat diseases.

## Data Availability

The original contributions presented in the study are included in the article/supplementary material. Further inquiries can be directed to the corresponding authors.

## References

[1] Radford AD Coyne KP Dawson S Porter CJ Gaskell RM. Feline calicivirus. Vet Res. (2007) 38:319–35. doi: 10.1051/vetres:2006056 17296159

[2] Cubillos-Zapata C Iván Angulo Almanza H Borrego Belén Bárcena J. Precise location of linear epitopes on the capsid surface of feline calicivirus recognized by neutralizing and non-neutralizing monoclonal antibodies. Veterinary Res. (2020) 51:59. doi: 10.1186/s13567-020-00785-x PMC7195702 32357948

[3] Sun Y Deng M Peng Z Hu R Chen H Wu B. Genetic and phylogenetic analysis of feline calicivirus isolates in China. Vet J. (2017) 220:24–7. doi: 10.1016/j.tvjl.2016.12.014 28190489

[4] Yang Y Liu Z Chen M Feng K Qi R Zheng Y. Classification of genotypes based on the *VP1* gene of feline calicivirus and study of cross-protection between different genotypes. Front Microbiol. (2023) 14:1226877. doi: 10.3389/fmicb.2023.1226877 37614595 PMC10442547

[5] Ellis TM. Jaundice in a Siamese cat with in *utero* feline calicivirus infection. Aust Vet J. (1981) 57:383–5. doi: 10.1111/j.1751-0813.1981.tb00527.x 6282244

[6] Lanave G Buonavoglia A Pellegrini F Di Martino B Di Profio F Diakoudi G. An outbreak of limping syndrome associated with feline calicivirus. Anim (Basel). (2023) 13:1778. doi: 10.3390/ani13111778 PMC10251824 37889723

[7] Pedersen NC Elliott JB Glasgow A Poland A Keel K. An isolated epizootic of hemorrhagic-like fever in cats caused by a novel and highly virulent strain of feline calicivirus. Vet Microbiol. (2000) 73:281–300. doi: 10.1016/S0378-1135(00)00183-8 10781727 PMC7117377

[8] Spiri AM. An update on feline calicivirus. Schweiz Arch Tierheilkd. (2022) 164:225–41. doi: 10.17236/sat00346 35232714

[9] Gao J Li Y Xie Q Al-Zaban MI Al-Saeed FA Shati AA. Epidemiological investigation of feline upper respiratory tract infection encourages a geographically specific FCV vaccine. Vet Sci. (2023) 10:46. doi: 10.3390/vetsci10010046 36669047 PMC9864582

[10] Mao J Ye S Li Q Bai Y Wu J Xu L. Molecular characterization and phylogenetic analysis of feline calicivirus isolated in guangdong province, China from 2018 to 2022. Viruses. (2022) 14:2421.doi: 10.3390/v14112421 36366519 PMC9696216

[11] Zhou L Fu N Ding L Li Y Huang J Sha X. Molecular characterization and cross-reactivity of feline calicivirus circulating in southwestern China. Viruses. (2021) 13:1812. doi: 10.3390/v13091812 34578393 PMC8473038

[12] Afonso MM Pinchbeck GL Smith SL Daly JM Gaskell RM Dawson S. A multi-national European cross-sectional study of feline calicivirus epidemiology, diversity and vaccine cross-reactivity. Vaccine. (2017) 35:2753–60. doi: 10.1016/j.vaccine.2017.03.030 28389099

[13] Smith SL Afonso MM Pinchbeck GL Gaskell RM Dawson S Radford AD. Temporally separated feline calicivirus isolates do not cluster phylogenetically and are similarly neutralised by high-titre vaccine strain FCV-F9 antisera *in vitro* . J Feline Med Surg. (2020) 22:602–7. doi: 10.1177/1098612X19866521 PMC7252219 31411533

[14] Di Martino B Marsilio F Roy P. Assembly of feline calicivirus-like particle and its immunogenicity. Vet Microbiol. (2007) 120:173–8. doi: 10.1016/j.vetmic.2006.10.021 17126499

[15] Percie du Sert N Hurst V Ahluwalia A Alam S Avey MT Baker M. The ARRIVE guidelines 2.0: Updated guidelines for reporting animal research. PloS Biol. (2020) 18:e3000410.doi: 10.1371/journal.pbio.3000410 32663219 PMC7360023

[16] Zhang L Xu W Ma X Sun X Fan J Wang Y. Virus-like particles as antiviral vaccine: mechanism, design, and application. Biotechnol Bioprocess Eng. (2023) 28:1–16. doi: 10.1007/s12257-022-0107-8 36627930 PMC9817464

[17] Sherwood J Mendelman PM Lloyd E Liu M Boslego J Borkowski A. US Navy study team. Efficacy of an intramuscular bivalent norovirus GI.1/GII.4 virus-like particle vaccine candidate in healthy US adults. Vaccine. (2020) 38:6442–9.doi: 10.1016/j.vaccine.2020.07.069 32878708

[18] Vesikari T Saez-Llorens X Blazevic V Lopez P Lopez E Masuda T. Immunogenicity of a bivalent virus-like particle norovirus vaccine in children from 1 to 8 years of age: A phase 2 randomized, double-blind study. Vaccine. (2022) 40:3588–96. doi: 10.1016/j.vaccine.2022.04.089 35595661

